# Metabolic Objectives and Trade-Offs: Inference and Applications

**DOI:** 10.3390/metabo15020101

**Published:** 2025-02-06

**Authors:** Da-Wei Lin, Saanjh Khattar, Sriram Chandrasekaran

**Affiliations:** 1 Center for Bioinformatics and Computational Medicine, Ann Arbor, MI 48109, USA; daweilin@umich.edu; 2 Department of Statistics, University of Michigan, Ann Arbor, MI 48109, USA; 3 Department of Biomedical Engineering, University of Michigan, Ann Arbor, MI 48109, USA; saanjh@umich.edu; 4 Program in Chemical Biology, University of Michigan, Ann Arbor, MI 48109, USA; 5 Rogel Cancer Center, University of Michigan Medical School, Ann Arbor, MI 48109, USA

**Keywords:** metabolic network, genome-scale metabolic modeling, transcriptomics, proteomics, metabolomics, metabolic objectives, archetypes, machine learning

## Abstract

**Background/Objectives:** Determining appropriate cellular objectives is crucial for the system-scale modeling of biological networks for metabolic engineering, cellular reprogramming, and drug discovery applications. The mathematical representation of metabolic objectives can describe how cells manage limited resources to achieve biological goals within mechanistic and environmental constraints. While rapidly proliferating cells like tumors are often assumed to prioritize biomass production, mammalian cell types can exhibit objectives beyond growth, such as supporting tissue functions, developmental processes, and redox homeostasis. **Methods:** This review addresses the challenge of determining metabolic objectives and trade-offs from multiomics data. **Results:** Recent advances in single-cell omics, metabolic modeling, and machine/deep learning methods have enabled the inference of cellular objectives at both the transcriptomic and metabolic levels, bridging gene expression patterns with metabolic phenotypes. **Conclusions:** These in silico models provide insights into how cells adapt to changing environments, drug treatments, and genetic manipulations. We further explore the potential application of incorporating cellular objectives into personalized medicine, drug discovery, tissue engineering, and systems biology.

## 1. Introduction

Cells must perform diverse tasks in order to survive, grow, and carry out specialized functions within multicellular organisms [1]. The specific objectives that dictate a cell’s behavior, resource allocation, and priorities are fundamental representations of cellular phenotypes in systems biology. Identifying and mathematically representing these objectives for different cell types is crucial for systems biology studies that aim to model and predict cellular processes holistically [2,3,4,5]. Assumptions about uniform cellular objectives, like biomass maximization, are often incorrect. They miss the complex links between gene expression, metabolic activity, and the different functions of specialized cell types [6,7]. Furthermore, evolutionary selection can promote phenotypic heterogeneity within cell populations. A better model of cellular objectives, under different regulations and environments, could improve phenotype predictions [8,9,10,11,12,13].

Techniques like flux balance analysis (FBA) rely on mathematically defined cellular objectives to predict metabolic fluxes and phenotypes using genome-scale metabolic models (GEMs) [14]. However, focusing solely on biomass production disregards the complex trade-offs and constraints that shape a cell’s metabolic strategies [15]. This oversimplification has spurred research to (i) evaluate common objective functions, (ii) infer new formulations, and (iii) create metabolic goals that are more biologically accurate [16].

**Figure 1:**
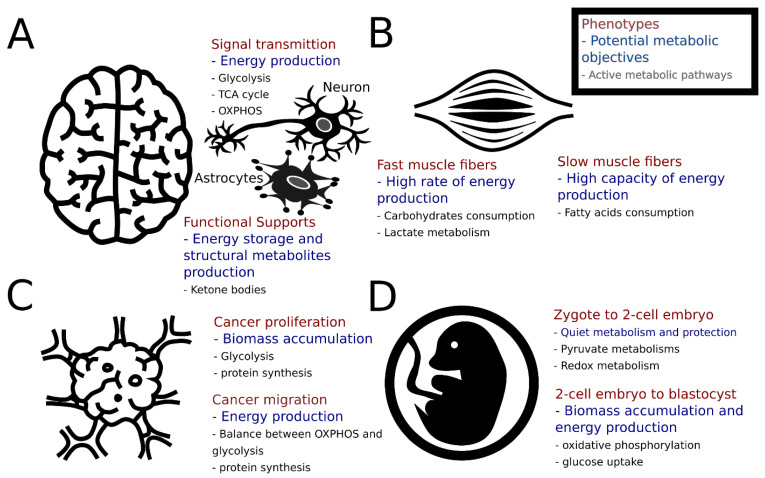
Cellular states, featured metabolic functions, and potential metabolic objectives. (**A**) Neurons and astrocytes are two types of brain cells. Neurons govern signal transmission, which requires active energy metabolism to produce ATP. On the other hand, astrocytes function as energy repositories. (**B**) Skeletal muscle mainly consists of fast and slow muscle fibers. Energy metabolism is required for both, but they consume different types of metabolites to support their functions. (**C**) Proliferation is considered the most important phenotype for cancer and tumors display aerobic glycolysis (Warburg effect). Although another phenotype, migration, relies on similar metabolic pathways, the relative allocation among various pathways is different from that seen in biomass synthesis. (**D**) Embryogenesis progressively changes cellular phenotypes from having low metabolic activities to high biomasses and energy production. The underlying design principle of this phenotypic change remains unclear.

Furthermore, the assumption of biomass production as the dominant objective for rapidly proliferating cells in systems biology studies may be reasonable for cancer cells and microbes undergoing rapid division, but is unrealistic for several other cell types (Figure 1). This assumption oversimplifies the nuanced objectives of non-proliferative cells like neurons, muscle cells, and embryonic stem cells, which often prioritize tasks beyond growth, such as tissue maintenance, developmental regulation, or the management of energy dynamics [17].

This review explores the metabolic and transcriptional facets of cellular objectives across diverse cell types. We emphasize the limitations of univariate growth assumptions and highlight computational methods that address the multi-objective nature of cellular priorities. By bridging transcriptomic archetypes with metabolic models, we aim to elucidate how cells coordinate gene expression and metabolism to modulate functions, maintain fitness within multicellular systems, and adapt to changing internal or external environments.

## 2. Metabolic Goals of Mammalian Cell Types

Several types of mammalian cells perform functions within multicellular systems that are not directly associated with single-cell fitness [18,19]. For instance, human cells are highly proliferative during development, driven by stem cells, but strict regulations turn most cells into quiescent states in adults [20,21]. The goals of these cells are the maintenance and execution of specific tasks for tissue functions, meaning that growth is no longer the primary objective.

Despite being mostly quiescent, the brain, muscle, and liver remain metabolically active, consuming 50% of oxygen, which implies that their metabolic objectives support the function of cells beyond growth [22]. For example, muscle cells tend to be quiescent until receiving proliferative signals, after which they differentiate to repair tissues [23]. During exercise, ATP production drives muscle contraction, with different types of muscle relying on different types of metabolism and different energy sources [24]. The metabolic objectives of muscle tissues can be heterogeneous and dynamic, depending on the type and intensity of the activity.

Brain cells prioritize oxidative and energy-related metabolism. This supports activity and prevents issues like hypoxia and cellular stress [25]. The brain consumes oxygen to fuel its electrical activity and support the functions of the nervous system, stabilizing membrane potentials through active ion transport [26]. Brain cell maintenance is vital for neuronal activity. Metabolites like oxygen and glucose are crucial for respiration, neurotransmitter synthesis, and cell communication [27].

Stem cells and embryos operate differently due to their unique cellular environment. During embryogenesis, the cellular program is highly regulated to ensure a healthy embryo. The quiet embryo hypothesis suggests embryos maintain low levels of protein synthesis, amino acid uptake, and glucose and oxygen consumption. This is to avoid damage and maintain stability [28]. During early development, embryos rely on maternal sources since they cannot produce their own biological materials [29,30]. This reliance highlights the importance of maintaining developmental potential rather than solely focusing on biomass growth.

Cancer cells typically prioritize growth to maintain proliferation. However, the presence of the Warburg effect—where aerobic glycolysis occurs with or without sufficient oxygen—suggests that a different metabolic strategy is used by highly proliferative cancers in various and noisy environments [31,32]. Pathogenic bacteria adopt a similar metabolic strategy when rapidly expanding within hosts [33,34,35]. Growth and division are common phenotypes associated with biomass accumulation. However, the specific cellular objectives can vary significantly based on the environment and the cell’s functional state. Various bioenergetic pathways drive the functions of cytoskeletons, including glycolysis, glutaminolysis, the pentose phosphate pathway, amino acid metabolism, lipid metabolism, and the maintenance of the redox state [36,37,38,39]. These functions contribute to the metastatic potential of various cancers and also play a role in cancer proliferation [40]. Studies suggest that the balance between aerobic glycolysis and oxidative phosphorylation, specifically the increase in aerobic glycolysis, may be more critical for cancer migration than proliferation [41,42]. The accumulation of the lactate generated from aerobic glycolysis is considered a driving force behind the invasiveness of cancers [43]. Therefore, primary and metastatic tumors may share similar metabolic objectives, but they fulfill them in different proportions.

Overall, unique metabolic objectives shape the metabolic characteristics of mammalian cells, challenging the usual focus on biomass objectives. The specific constraints imposed on various cell types underscore the need to investigate the intersection between cellular metabolism and developmental direction.

### 2.1. Cellular Objectives, Trade-Offs, and Archetypes

**Figure 2:**
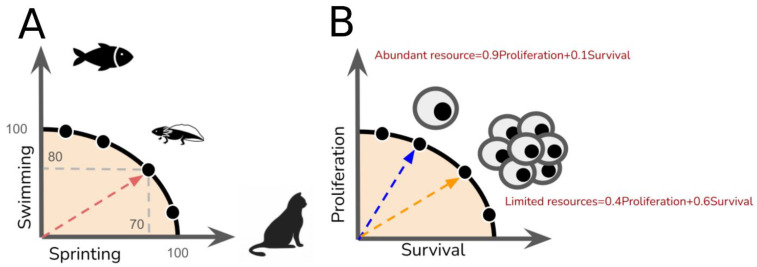
The Pareto optimality and trade-offs of biological phenotypes. The trade-offs involved in optimizing a pair of objectives can be represented by 2D Pareto fronts. (**A**) Fins and legs are biological phenotypes optimized for two distinct objectives—swimming and sprinting, respectively. A fish and a cat serve as metaphors for archetypal species, or single-objective species, that have optimized their performance for swimming or sprinting. In contrast, the axolotl represents a multi-objective species attempting to optimize both swimming and sprinting simultaneously. However, a trade-off forces the axolotl to optimize these two objectives along the Pareto front; as a result, its swimming and sprinting performance is only 80% and 70% relative to that of the fish and cat, respectively. (**B**) Cancer or microbial populations face trade-offs between survival and proliferation. The choice of phenotypes is hypothetically determined by the abundance of resources.

While cells aim to achieve various objectives, it is nearly impossible to optimize all of them at once, which leads to trade-offs [44]. A trade-off is an idea widely studied in evolutionary biology [45]. For example, no type of species has been found that can run as fast as a cat and swim as smoothly as a fish at the same time (Figure 2A). Running and swimming are biological traits that species tend to optimize as key life history objectives, which are critical for survival and reproduction. However, realistic limitations, such as the finite energy available for respiration systems, constrain the design of biological systems. Cellular objectives are hence shaped by resource allocation constraints [46]. Extreme resource allocations, known as archetypes, maximize specific functions but may reduce resilience or adaptability [47], which are essential for buffering noise and responding to environmental changes. For example, cancer cells often perform multiple tasks due to their heterogeneity. In contrast, normal tissues have a structured organization with archetypal goals [48,49,50].

A classic conceptual model called the Y-model depicts two phenotypes competing for limited resources [51]. The Y-model, serving as a fundamental theory, has guided researchers to study pairs of phenotypes with top-down methods like hormone manipulation over a phenotype [52]. However, the formation of biological trade-offs is more intricate [51]. They exist both inter- or intra-species and can be hidden by different environmental conditions [45].

The Y-model is applied to develop FluTO, which hypothesizes that trade-offs among metabolic reactions can be mathematically described by the equation $Y=\Sigma \alpha_{i}x_{i}$. In this model, Y represents a common resource, formulated as a linear combination of traits $x_{i}$, where each trait is weighted by a coefficient $\alpha_{i}$, which determines the allocation of Y. Based on this assumption, FluTO uses flux variability analysis (FVA) to determine invariant reaction fluxes under specific boundary conditions, with Y serving as the resource constraint. FluTO then designates a weighted sum of fluxes equal to an invariant flux, identifying absolute trade-off fluxes that depend on the available carbon sources in *Escherichia coli* and *Saccharomyces cerevisiae* [53]. An adaptive version, FluTOr, was later developed to identify relative trade-offs, where the resource Y is variable, allowing for phenotypic plasticity [54].

Similar ideas have been applied in large-scale models, suggesting that a trade-off is widely present in various phenotypes [55]. For example, the expression of growth and survival genes in *Escherichia coli* cannot be optimized at the same time. The expression of growth was active in the exponential phase, despite a change in culture conditions. In the stationary phase, survival genes were active [55]. Although the research did not directly investigate the survival and growth trade-offs during metabolism, it confirmed that Pareto optimality exists in the phenotype space. These studies focused on extreme cases known as ‘archetypes’, which served as reference points. Specifically, the two archetypes represented growth-optimized and survival-optimized *Escherichia coli*. As the transition occurred from the growth-optimized to the survival-optimized strain, individuals proportionally optimized these two objectives—growth and survival (Figure 2B). Mathematically, the performance in these objectives can be represented by a Pareto front, where any individual hypothetically allocates their resources to the objectives within this front. Similarly, since microbial species must adapt to rapidly changing environments, other studies have also suggested that trade-offs exist between the growth rate and other objectives, such as adaptation [56], survival [57], and mobility [58].

**Figure 3:**
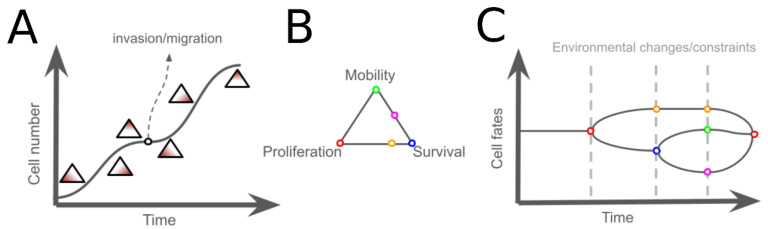
The switch of cancer phenotypes in relation to trade-offs and environmental changes. Inspired by Aktipis et al., a model for the switching of phenotypes during the expansion of a cancer population is shown. (**A**). The dynamics of cancer phenotypes are governed by trade-offs among proliferation, survival, and mobility (**B**). In the lag phase, cells tend to optimize proliferation. As the population reaches the stationary phase, the focus shifts toward optimizing survival. When the population ceases to grow and resources like oxygen become depleted, mobility is optimized (**A**). The switching of cell phenotypes can be characterized by bifurcation (**C**).

Interestingly, similar trade-offs are observed in cancers. Aktipis et al. reviewed studies of cancer phenotypes and summarized that cancers push the optimization of proliferation and survival simultaneously toward Pareto optimality until they exhaust limited resources. In other words, a trade-off emerges between proliferation and survival within a cell population [59]. However, Hausser et al. argued that spatial and temporal variations create selection pressures that force cancer cells to switch phenotypes [60]. For example, late-stage cancers tend to optimize survival under hypoxic conditions, contrasting with early-stage cancers that are proliferation-optimized due to ample oxygen availability, as depicted in Figure 2B [61]. Spatially, environmental niches, such as when proximity to blood vessels affects nutrient and oxygen supply, can also lead to trade-offs between proliferation and survival phenotypes [62,63]. Although this trade-off influences the selection of cancer phenotypes in response to the environment, Aktipis et al. noted that cell migration, specifically cancer invasion, enables cancers to dynamically optimize either proliferation or survival. In fact, proliferation and migration have been reported to exist as a pair of phenotypes that potentially form a trade-off in cancer [64], implying that more complicated interactions among objectives construct a high-dimensional trade-off embedded in the evolution of cancer (Figure 3A,B). Changes in constraints or resources can lead to the bifurcation of cancer phenotypes driven by these trade-offs (Figure 3C). Although bifurcation theory is beyond the scope of this review, the way bifurcation interacts with the biological trade-offs is worth discussion in future studies [65,66]. Given that trade-offs can consist of multiple objectives in reality, leveraging genomics data has become a promising direction for studying the interactions among objectives, trade-offs, and archetypes at the cellular level.

### 2.2. Brief Overview of Metabolic Modeling and Flux Balance Analysis (FBA)

The FBA approach simulates genome-scale metabolic network models (GEMs) to predict reaction fluxes based on objective functions and constraints. GEMs are the mathematical representation of the interconnection of chemical reactions, metabolic enzymes, and genes [67]. Numerous studies have taken advantage of the flexible mathematical nature of GEMs and have used it to interpret transcriptomics [67,68,69,70,71,72,73], proteomics [74,75], metabolomics [76], and epigenomics datasets [77,78,79,80]. FBA applies various constraints from omics and thermodynamics to optimize the flux distributions through metabolic networks through linear programming (Equation (1)).

(1) $$max\;Z\;\;=\;c^{T}v\;s.t.{\{}_{Sv=0}^{v_{lb}<v<v_{ub}}$$

In Equation (1), *Z* is the objective function; *c* is the vector of weights, indicating the contribution of each reaction to the objective function; and *v* is the vector of reaction fluxes. Flux balance analysis uses two additional constraints. The first constraint is that the product of the fluxes and stoichiometric matrix should be zero, which represents a steady state condition shown by *Sv = 0* (Equation (1)). Here, the stoichiometric matrix is a mathematical representation of the interlinked metabolic reactions. The matrix bypasses the need for kinetic parameters, which are unknown, and the model does not exhibit dynamic behavior. The second constraint describes the constraints on the solution spaces through the upper and lower bounds of each reaction. FBA uses linear programming techniques to find the best flux solution for the optimization function. Biomass is widely used as the optimization objective in order to model proliferative cells, including bacteria, yeast, and cancers. However, maximizing biomass production can be counterintuitive when modeling non-proliferative cells, such as adult brain cells [17].

### 2.3. Refining Biomass Objective Functions

Biomass objective function (BOF) is the most commonly used objective and consists of essential metabolites, representing the growth and division of cells [14]. Mathematically, BOF is a linear combination of amino acids, nucleotides, lipids, and other growth-related metabolites, and it has been successfully used to model the proliferation and growth rates of many cell types [81,82,83,84,85]. Determining which metabolites should be involved and how their coefficients can be applied to individual cells is a research challenge [4,14].

The mathematical equation of BOF is often formulated during the network construction procedure. For instance, the SEED model presents an automated model reconstruction approach to GEMs. SEED helps to draft GEMs to predict organism phenotypes from genotypes and translate enzymatic processes into quantitative predictions [86]. The SEED framework helps to increase the rate of new model development and the number of sequenced genomes corresponding to metabolic models. The model translated the gene annotations into a metabolic reaction list via the annotation ontology map, and provided template biomass equations for 130 organisms [86].

In addition to the genotype, different growth conditions and cell types may lead to different BOFs. Beck et al. 2018 designed a protocol to experimentally measure and select macromolecules (carbohydrate, DNA, lipid, protein, and RNA) for biomass synthesis, considering culture conditions. Their condition-specific biomass objective functions can predict biomass production best in three different bacterial species [87]. Furthermore, the coefficients of metabolites in biomass objectives can affect growth prediction. Nevertheless, the changes in biomass objective coefficients are notably less crucial in the assessment of cancer gene essentiality compared to the network structure and the gene-protein-reaction rules [8].

**Figure 4:**
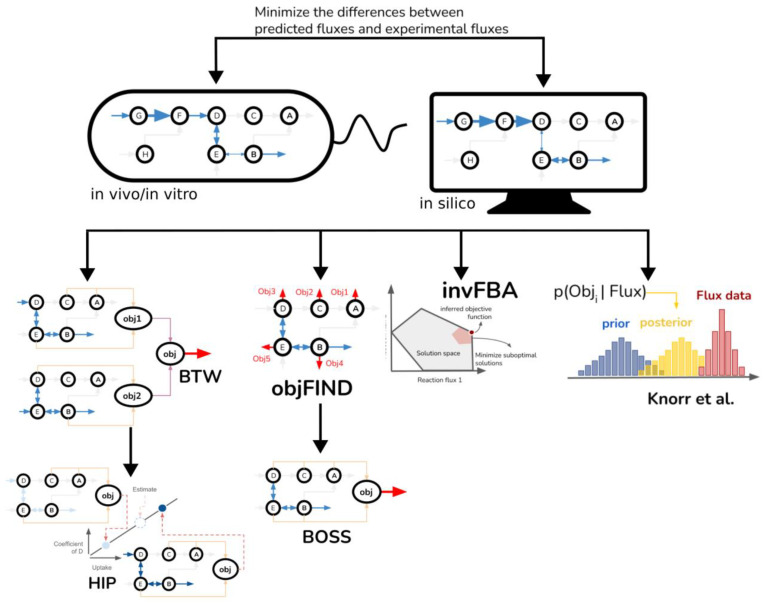
Methods leveraging experimentally measured reaction fluxes to infer or refine objective functions. The methods illustrated share a common principle: minimizing the difference between experimental fluxes and simulated fluxes within their optimization frameworks. However, approaches to handling draft objective functions differ. Knorr et al. rank candidate objectives, invFBA identifies the smallest suboptimal space, objFIND assigns weights to metabolites, and BOSS randomizes the coefficients of metabolites. For BTW and HIP, experimental flux data—specifically uptake fluxes and growth rates—are utilized. BTW combines multiple draft objectives with equal weights to define its objective function, while HIP adjusts the coefficients of biomass precursors dynamically based on changes in uptake rates [88,90,91,92,93].

To address the uncertainty of biomass objectives in different environmental conditions, Biomass Tradeoff Weighting (BTW) and higher-dimensional-plane interpolation (HIP) approaches can be used. They identify biomass objectives with consideration of environmental changes that are reflected in exchange reactions [88]. BTW incorporates multiple biomass equations into a model by weighting their coefficients to fit the maximal growth. In contrast, HIP assumes linear relationships between environmental changes and biomass compositions, thus applying interpolation to approximate the coefficients of metabolites in the biomass equation with experimental data about environmental changes (Figure 4). The research emphasizes the influences of the environment on the biomass objectives. Instead of inferring or summarizing single biomass objectives, the pFBAwEB pipeline alternatively considers the variation in the biomass compositions of different cells and assesses possible ranges of coefficients in biomass objectives [89]. This work identifies DNA and fatty acids as the only two groups of metabolites with low variation (Figure 4).

Despite the success of BOF, studies have found that maximizing energy, such as in ATP production, aligns better with physiological conditions [94,95,96]. Evolutionary optimization models have shown that the metabolic networks optimized for the production of ATP and NADH have similar structural designs in terms of glycolysis [97,98]. Another study alternatively leveraged the optimization of NADPH production as the objective function to successfully model the metabolic states of NCI-60 cancer cell lines [99]. However, a rich culture medium was better predicted via non-linear optimization of ATP production [6]. That being said, Schuetz et al. suggested that combining two or more objective functions was required to estimate reaction fluxes with better validation outcomes. This accentuates the necessity of the calibration of objective functions or the employment of multi-objective functions in specific conditions.

Unlike studies on cancer or pathogens that are modeled by optimizing biomass, studies on aging and lifespan utilize alternative objective functions. In this case, the sequential optimization of objective functions was used, including maximal biomass production, non-growth-associated maintenance, and ATP production [100]. Maximal biomass production was found to be essential to prolonging the lifespan in yeast, which could be extended by optimizing non-growth-associated maintenance as the second objective. In addition, potential trade-offs were introduced in another study where aging, reproduction, and growth competed with each other when optimizing their values [101]. The balance of multiple objectives might be essential to optimizing yeast fitness.

### 2.4. Multi-Objective Frameworks for Predicting Metabolic Behaviors

The trade-off between multiple objectives can be described by Pareto optimality. For example, the microbial growth rate forms a trade-off with the yield of reaction fluxes [102,103,104]. This trade-off reveals that although high-yield fluxes require larger amounts of enzymes, the protein production burden of those enzymes results in the decreased growth of cells. A previous study suggested that the maximization of ATP and biomass and the minimization of total fluxes competed with each other quantitatively [6]. Intriguingly, the authors found that microbial species allocated their objective fluxes close to the level of Pareto optimality. Nonetheless, the distance between the fluxes and the Pareto optimality is still remarkable compared to the flux allocations in different conditions, such as switching from aerobic to anaerobic metabolisms. The study concluded that the distance of the fluxes from the Pareto optimality allowed them to maintain the flexibility to quickly respond to environmental changes [6]. In other words, cells would rather sacrifice optimality to minimize flux adjustment efforts than change their metabolic objectives [6]. Inspired by these studies, parsimonious FBA (pFBA) improves the accuracy of metabolic modeling by considering the enzyme costs as an additional objective function [105]. Mathematically, pFBA solves a multi-objective problem by maximizing the biomass objective and minimizing the sum of reaction fluxes.

In bioproduct production, the goal is to maximize the production of valuable chemicals in bacterial, yeast, or mammalian cell factories. However, the production of the target chemicals impedes the growth rates of cells, which ultimately reduces the total product yield. Thus, both the growth rate and the production of the target chemicals should be optimized. The OptKnock framework is an example that optimizes bioproduct yield by coupling it with biomass fluxes [106]. OptKnock relies on a bi-level nested linear programming problem. It maximizes chemical production, subject to maximizing biomass production (an FBA problem).

A related method is multi-objective metabolic mixed-integer optimization (MOMO). Inspired by Pareto optimality, MOMO was designed by treating the production of target chemicals and biomass as a trade-off [107]. In other words, MOMO maximizes biomass and target chemicals simultaneously, instead of solving a bi-leveled linear programming problem with maximized biomass as a priority. The algorithm minimizes the differences between the fluxes of knockout and wild-type strains, which is derived from the idea of minimum flux adjustment in knockout strains. The integration of evolutionary algorithms can solve complex multi-objective problems in large networks. Methods like OptGene and multi-objective metabolic engineering (MOME) use genetic algorithms (GAs) to find the best knockout strains for growth and biochemical production [108,109]. These methods are able to suggest the best knockout strain for lactate production while maintaining cell growth.

### 2.5. The Inference of Metabolic Objective Functions Based on Experimental Fluxes

Knorr et al. applied Bayesian statistics to determine the objective functions in *E. coli* in relation to a set of experimental fluxes (Figure 4). The likelihood of a function serving as a true objective could be estimated by comparing the deviation of predicted fluxes from the experimental data. The authors prepared 5 candidate objective functions, including the maximum growth rate, the minimum redox potential, maximizing or minimizing ATP production, and the minimum nutrient uptake. Surprisingly, minimizing the production rate of redox potential eventually achieved the highest probability as an objective function for *E. coli* growing on succinate. The function of the maximizing growth rate also predicted acetate production closer to experimental data [93]. A drawback of this method is that it requires candidate objective functions. Another drawback is that each candidate function is evaluated individually, which implies that the linear or non-linear combination of the candidate functions is not considered.

The Biological Objective Solution Search (BOSS) method infers the objective function of biological systems from the network’s stoichiometry and experimental fluxes [91]. Conceptually, BOSS solves a bi-level optimization problem. The goal is to minimize the sum-squared error between experimental fluxes and in silico fluxes subjected to an FBA problem. Mathematically, the bi-level optimization problem is transformed into a single-level problem with the duality principle. A putative objective function is selected from a pool of randomly generated objective functions, and the putative functions are then clustered based on similarity. The largest cluster is eventually chosen as the most common objective. BOSS successfully estimated that precursor biomass synthesis reaction was the objective of yeast central metabolism.

BOSS builds upon ObjFIND, which minimizes the distance between the experimental flux data and flux distributions when optimizing a hypothetical objective function [90]. Instead of creating a new reaction in BOSS, ObjFIND utilizes coefficients of importance (CoIs) to explain the experimental flux data, quantifying the fraction of the additive contribution of a given flux to an objective function. A greater CoI represents a higher degree of importance of the flux, in which the fraction of each flux is consistent with the experimental flux data. Interestingly, CoIs of either aerobic or anaerobic growth conditions were found to be similar whereas their flux distributions were different. ObjFIND determines how likely a reaction is likely to be a component of an objective function. In contrast, BOSS defines the objective function as an additional reaction in the stoichiometric matrix and can avoid choosing objective functions from suboptimal solutions, but the non-convex problems may lead to local minimum solutions.

Given the non-convex problems in ObjFIND and BOSS, inverse flux balance analysis (invFBA) can ensure global optimality and polynomial computation when looking for objective functions [92]. Specifically, invFBA assumes the existence of noises in experimental flux measurements. The assumption leads to the first step of invFBA that minimizes the errors between modeled and observed fluxes. Since the objectives that generate the near-optimal fluxes may not be unique, the L1 norm is applied in the second step to narrow down a set of candidate objectives. In the third step, the objective coefficients are set to be sparsest, which is equal to fewer non-zero coefficients in the objective. In terms of the performance of invFBA in toy models, the algorithm successfully recovered the biomass objectives in three culture conditions. Additionally, FBA-optimizing alternative objectives, including the maximization of ATP synthase and the minimization of glucose uptake, were also captured by invFBA. A study validated the invFBA approach by experimenting with time-dependent fluxes from gene expression data of *S. oneidensis* and fluxes within the central carbon of *E. coli* [92]. invFBA showed the strains optimizing respiratory efficiency in addition to their growth rates, providing insight into the species’ metabolic rate through the depletion of a carbon source. Computationally, invFBA only seeks to solve objective functions by linear programming, which guarantees computational efficiency and global minimums of the solutions.

### 2.6. The Inference of Metabolic Objectives from Genomics Data

Flux data have been effective in inferring objective functions in bacteria and yeast. However, the lack of fluxomics in most bacterial species and eukaryotic cells impedes the application of the methods mentioned in the last section. For example, even with invFBA’s expansion of metabolic modeling and the success of identifying the flux’s objective function, the method is limited due to the insufficiency of fluxomics in terms of enabling invFBA to distinguish between similar metabolic reactions [92]. As a whole, genomics, including transcriptomes, proteomes, and metabolomes, can provide evidence and constraints to narrow down the range of putative metabolic objectives.

One such algorithm that addresses this is the biomass objective function algorithm (BOFdat), which pulls from experimental data and utilizes a standardized computational platform to determine the species-specific biomass objective functions of various species [110]. BOFdat takes an unbiased, data-driven approach. The algorithm consists of three independent categories: major macromolecules, coenzymes and inorganic ions, and species-specific metabolic end goals. After defining the candidate metabolites from the three categories, BOFdat seeks the best metabolic objective with which to predict gene essentiality using a genetic algorithm (GA). Specifically, each generation of genetic algorithm performs the muting and mating of the objective functions that can better predict gene essentiality. Since every candidate metabolite in GA only has binary values, the objective function generated by genetic algorithms represents the necessity of predicting cell growth. BOFdat was used to reconstruct the biomass objective functions of *E. coli* and it outperformed BOSS and SEED in terms of the phenotypic predictions. Additionally, BOFdat was also employed to estimate objective functions in cancer cells and improve predictions of drug targets [8].

There are two key limitations of BOSS. First, genetic algorithms may result in unrealistic objectives if they fail to account for network structure, even though they can still predict gene essentiality effectively. Essential genes predicted with BOFdat-based objectives were suspected to be overestimated due to the identification of uncommon genes [8]. Second, BOFdat relies on omic datasets, growth rate measurements under various conditions, and the assessment of growth rate changes in knockout strains to identify essential metabolites. The association between omics data and growth largely determines how BOFdat summarizes the metabolic goals of organisms. BOFdat might not fit slowly growing cells or terminally differentiated cells in the muscle and brain.

Numerous methods have been developed to tackle the uncertainty inherent in cellular objective estimation. However, there is no consensus regarding the preferred approach. This challenge is especially pronounced when dealing with GEMs of mammalian cells, where the larger number of metabolites and metabolic genes introduces complexity. Moreover, the interrelated nature of metabolic networks means that objective values are indirectly shaped by the setup of gene–protein reaction (GPR) rules in GEMs and the environmental conditions [8]. Consequently, a comprehensive evaluation of various objective functions and GEMs becomes imperative for leveraging metabolic modeling in drug discovery.

### 2.7. Beyond Metabolism: Discovery of Cellular Objectives with Gene Expression Profiles

Integrating transcriptome-level objectives with metabolic models could provide a more comprehensive view of how gene expression patterns interface with metabolic activity to determine a cell’s functional priorities. Data-driven approaches can use transcriptomic profiling to predict lineage trajectories and “archetypes” that represent dominant cellular programs or extreme phenotypic states [111,112,113,114,115,116,117].

A transcriptional change can be connected with trade-offs among objectives associated with biological traits [55]. Given that biological traits cannot be optimized simultaneously, the traits form trade-offs or Pareto fronts. Schuetz et al. relied on Pareto fronts to build a scalable theoretical framework called the Pareto task inference method (ParTI) [116]. ParTI assumed that all phenotypes fall into a trait space formed by transcriptomics observations. The method includes two parts. First, deep learning methods are used to extract features from transcriptomics data that can represent certain biological tasks. Second, archetypical computing relies on the features to infer the most important biological tasks, known as archetypes. ParTI has been applied to many biological systems. In cancers, ParTI identifies biological tasks for five types of cancers and determines the differential sensitivities to drugs according to the perturbations of the tasks [118].

There is a spatial division of labor in tissues. For example, the cells positioned at the center of the liver have distinct metabolic activities compared to the cells at the edge. Based on this idea, Adler et al. extended the ParTI framework by adding a spatial dependent function. Adler et al.’s integrated method reported spatial patterns of biological tasks in the intestinal villus and liver hepatocytes that were not clearly shown with t-SNE [119]. Interestingly, their work found that the assumption of the crosstalk between archetypes increased the predictability of spatial patterns that mimic cell–cell communication in tissues. For example, local inhibition between archetypes can represent Delta–Notch interactions in mouse colon fibroblasts [120].

Modeling the transitions of phenotypes or biological tasks provides quantitative evidence of adaptation to selective pressures, such as drug treatments. Another study leveraged ParTI to analyze longitudinal transcriptomics during therapy. It identified three archetypes—metabolism, cell defense, and DNA repair—that led to resistance in high-grade serous ovarian cancer [121]. A related study identified 12 immune archetypes across pan-cancers using only 10 different compositional features, such as CD4+ cells [122]. Clustering cancers based on these pre-selected features helps to explain the immune functions observed in pan-cancers. An analysis of transcriptomes linked to the 12 archetypes found that the genes were enriched in tumor proliferation, diverse tumor transcriptome programs, and overall survival. The study heavily relied on experimental results to demonstrate that the heterogeneity of transcriptomic states among archetypes did not stem from tissue origins but rather choices in terms of immune functions [122]. Weistuch et al. also hypothesized that the cancer heterogeneity results from the trade-offs. They developed a method to approximate the archetypes relying on non-negative matrix decomposition (N-NMF) because the count of RNA-seq data involves non-negative values. Mathematically, a matrix of the gene expression levels of samples can be separated into expression levels using archetypes multiplied by archetypes multiplied by samples. This extracts hidden information about archetypes. Learning from the Genotype Tissue Expression (GTEx) data, which contain gene expression levels of normal tissues, they uncovered six archetypes. The same procedure revealed that drug sensitivities and mutated genes in the Cancer Cell Line Encyclopedia (CCLE) were significantly associated with the six archetypes. For example, the anti-cancer drug Irinotecan is associated with archetype 2, which is enriched in DNA repair, and mutated genes are sensitive to topoisomerase inhibitors [123].

**Figure 5:**
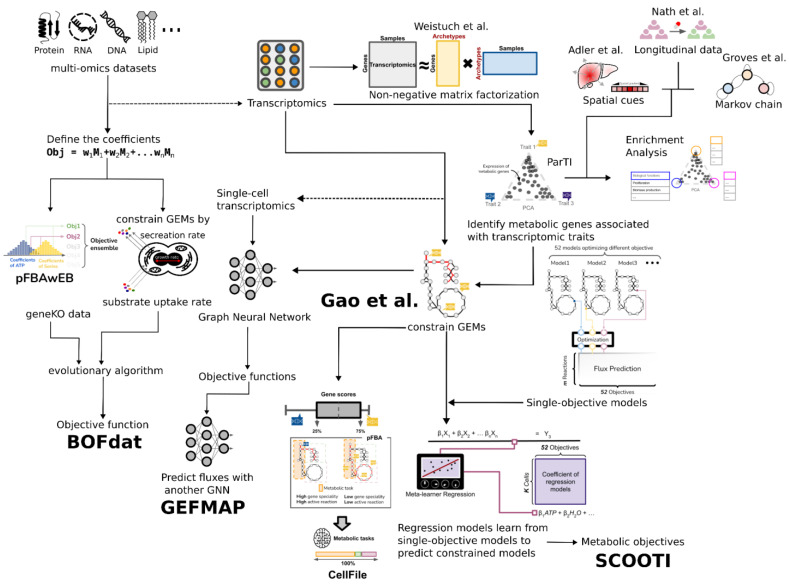
Methods leveraging genomics data to infer cellular objectives. An overview of methods used to infer cellular objectives and archetypes based on transcriptomics data is shown as an example. The flow chart shows shared features, similar inputs/outputs, or common techniques used in different methods. While the methods on the top-right corner only suggest cellular archetypes/objectives in transcriptional levels, the other methods are all capable of deriving metabolic objectives. It should be noted that transcriptome but also proteome and growth rate data are required for BOFdat [89,110,116,117,119,121,126,127,128,129].

Non-linear archetypal analysis of the transcriptome assumes the existence of a non-linear link between archetypes and gene expression. It can identify extreme biological functions. It can also group single cells to represent distinct states based on scRNA-seq and transposase-accessible chromatin sequencing (scATAC-seq) data. Single-cell aggregation of cell states (SEACells) leverages a Gaussian kernel to capture non-linear relationships between cells using similarity graphs of single cells [114]. The SEACells mtehod then employs archetypal analysis to group cell states based on the kernel matrix. This method effectively addresses the sparsity of single-cell data while maintaining sufficient resolution to recover cellular phenotypes, such as healthy and COVID-19-enriched cells. However, SEACells does not focus on the biological meanings of archetypes; instead, it uses archetypes as references to group single cells. In contrast, scAANet introduces an autoencoder to single-cell transcriptomics, mapping them to a latent space of archetypes. Similar to the ParTI method, scAANet can identify biologically meaningful archetypes of gene expression profiles and enhance downstream analyses, such as gene set enrichment analysis [115]. The key concepts of these methods are summarized in Figure 5 and Table 1.

While these methods are useful for identifying the goals different cells aim to achieve, considering only gene expression profiles may not accurately determine cellular objectives. In fact, assuming the existence of imperfect correlations between the transcriptional and enzymatic activities improves the predictive power of metabolic modeling [124]. Thus, trade-offs between metabolic pathways might not be represented in transcriptomes [53,125].

**Table 1:** Methods to infer transcriptomic objectives and archetypes.

Method	Input	Output	Summary	Study	Ref.
ParTI	Transcriptome	Archetypes	The computational framework relies on principal convex hull algorithms to discover archetypes from transcriptomics data.	Both microorganisms and cancer cells	[116]
ParTI+spatial gradient model	Transcriptome	Archetypes	A spatial gradient of performance was formulated based on ParTI.	Multicellular/tissue	[119]
ParTI+Markov chain models	Single-cell transcriptome and RNA velocity	Archetypes	Probabilistic transitions between parts based on ParTI to retrieve sequential dependencies between parts.	Cancer	[117]
ParTI+longitudinal analysis	Longitudinal single-cell transcriptome	Archetypes	The method was adapted from the ParTI framework to identify archetypes of ovarian cancer and how they evolved after treatments and therapies.	Ovarian cancer	[121]
Clustering with knowledge-based features	Transcriptome	Archetypes	Uses domain knowledge and clustering algorithms to identify archetypes.	Pan-cancer study	[122]
N-NMF	Transcriptome	Archetypes	Decomposes a non-negative matrix into two lower-rank matrices representing parts and features.	Cancer and drug discovery	[123]
SEACells	scATAC-seq and scRNA-seq	Metacells/archetypes	SEACells leverages archetypal analysis and an adaptive Gaussian kernel to identify archetypes based on similarity matrix of estimated single-cell data.	Hematopoietic differentiation and COVID-19 samples	[114]
scAANet	Single-cell transcriptome	Archetypes	Utilizes an autoencoder with a count distribution-based loss to extract gene expression profiles (GEPs) of archetypes and infer their relative activity across cells.	Samples of pancreatic islet, lung IPF, and prefrontal cortex	[115]

### 2.8. Integration of Pareto Framework, Multiomics and GEMs to Define Metabolic Objectives

Evolutionary biology aspects are commonly considered in metabolic modeling methods such as pFBA and MOMA. Gao et al. applied multi-objective genome-scale metabolic models to identify the metabolic tasks executed in TCGA cancer cells [129] (Figure 5). The computational framework was based on the Pareto task inference method (ParTI) [116]. ParTI assumed that all phenotypes fall into a trait space formed by transcriptomics observations. Using ParTI, Gao et al. identified important metabolic tasks that competed with each other in cancer subtypes. For instance, the luminal B subtype of breast cancer has significant metabolic tasks, including steroid synthesis and oxidative phosphorylation. The authors then mapped the significant metabolic tasks to GEMs and predicted reaction fluxes. Coupling with flux minimization served as the new objective function, and the method outperformed metabolic modeling methods with the maximization of biomass production as the objective function used to predict gene essentiality. A related study developed CellFile, which summarizes tissue- and cell-specific metabolic tasks using context-specific models based on transcriptomics datasets [128]. Reactions associated with a certain metabolic task were calculated with fluxes in context-specific models. Since the series of studies also carefully addressed the bias of constraint-based modeling methods based on transcriptomics, the metabolic tasks identified by CellFile could account for essential genes and crucial metabolic pathways in different cells.

While these methods are based on the expression levels of metabolic genes, post-transcriptional and post-translational regulation may modify the association between transcription and metabolism [80]. In addition, the biomass objective function was required in the method developed by Gao et al. and CellFile, leading to the inevitable assumption of cell growth.

To avoid the issue and answer the metabolic goals for cells with different proliferative abilities such as quiescent cells, a method called Single-Cell Optimization Objective and Trade-off Inference (SCOOTI) was developed. SCOOTI formulates cell-type-specific and cell-specific metabolic objectives, considering the optimization trade-offs seen in metabolism [126]. SCOOTI accurately differentiated the objectives of quiescent and proliferative cell types. It also correctly inferred metabolic objectives during different phases of the cell cycle. This shows that individual cells do not optimize all biomass components at the same time. Mathematically, SCOOTI uses regression models to fit a combination of flux vectors optimizing each metabolite to predict condition- or cell-specific fluxes. The coefficients estimated from the regression models are used to formulate cell-specific metabolic objectives. SCOOTI separated cell types in single-cell datasets by pursuing metabolic objectives, and identified trade-offs in cell states. For example, it uncovered trade-offs in redox metabolism and biomass synthesis, which explained the metabolic properties of distinct cell states during embryonic development. This trade-off was also seen during the cell cycle and may explain the division of metabolic tasks during cell cycle phases.

**Figure 6:**
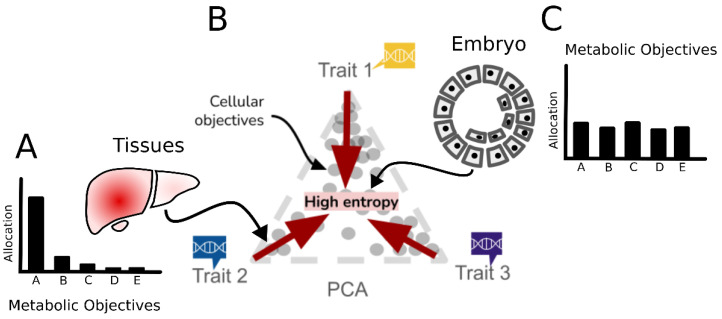
The use of entropy to summarize the resource allocation of cellular objectives. (**A**) A three-dimensional trait space, for example, theoretically shows higher entropy at the center of the triangle away from the archetypes. (**B**) When we project the metabolic objectives of tissues and embryos onto the trait space, the data points of the embryo are expected to be located closer to the center and display higher entropy. (**C**) In contrast, tissues or differentiated cells are expected to hypothetically stay away from the center and display lower entropy.

SCOOTI was applied to analyze cellular objectives during embryogenesis and measure uncertainty in metabolic tasks using Shannon entropy. Early-stage cells (zygotes to 1-cell) exhibited higher entropy, reflecting diverse metabolic tasks, compared to later stages (2-cell to blastocyst), which aligned more closely to archetypes (Figure 6). This suggests a link between objective entropy and cellular organization: differentiated cells optimize for specific tasks, while more pluripotent cells diversify their metabolic functions. These findings indicate that entropy could serve as a metric for pluripotency and cellular differentiation [130]. However, current entropy calculations are simplified and could benefit from approaches that consider objectives or cell interactions, like the maximum-entropy methods used in genomics and metabolic modeling [131,132].

Energy landscapes provide another perspective on cellular objectives. Hopfield networks, based on recurrent neural networks, model how systems settle into stable states (attractors) [133]. Deeper energy wells represent stable biological states (e.g., adult cells), while shallow wells reflect flexibility for differentiation (e.g., pluripotent cells) [134]. The mapping of cellular objectives to energy landscapes can quantify the stability of states and their transitions under perturbations, such as resource changes or drug treatments. For example, during embryogenesis, transitions between cell types can be understood by tracking shifts in energy wells. Integrating omics data with Hopfield networks enables predictions of cellular responses to environmental changes, offering insights into adaptation and robustness. By combining entropy, Hopfield networks, and systems biology, researchers can better understand how cells allocate resources, balance trade-offs, and maintain optimized functions. These tools open new possibilities for advancing bioengineering and medical interventions.

Cellular phenotypes, such as differentiation, development, migration, and apoptosis, are not only linked to cellular or tissue functions, but also to individual fitness. Future studies can align cellular phenotypes with inferred metabolic objectives to elucidate how and why cells modulate their metabolic functions to alter phenotypes. Leveraging trade-offs and Pareto analysis in multi-omics data (i.e., a combination of the concept of ParTI and SCOOTI) may help to uncover the driving forces that lead cells to adjust their metabolism to maintain a particular phenotype. In the long term, associating inferred metabolic objectives with gene regulatory and signaling networks can introduce “controls” into metabolic trade-offs, potentially providing biologically meaningful insights into fitness within multicellular systems.

All the methods share a similar concept, i.e., minimizing the difference between experimental fluxes and simulated fluxes in their optimization problems. Draft objective functions, however, are treated differently in the methods. While Knorr et al. only ranks candidate objectives, invFBA looks for the smallest suboptimal space, objFIND weighs metabolites, and BOSS randomizes the coefficients of metabolites. Uptake fluxes and growth rates are the experimental flux data required for BTW and HIP. BTW sets the objective function to the combination of multiple draft objectives with equal weights, whereas HIP interpolates the coefficients of biomass precursors with changes in uptake rates. GEFMAP differs from other methods by inferring metabolic objectives using graph neural networks (GNNs) [127]. It assumes that interconnected and highly active reactions are strong candidates for metabolic objectives. However, GEFMAP does not estimate objectives for individual conditions or single cells, as SCOOTI does.

SCOOTI was primarily designed to estimate metabolic objectives during cell-type transitions. In this regard, SCOOTI addresses the heterogeneity and uncertainty of metabolic objectives at single-cell resolution, a capability not present in other methods. Additionally, SCOOTI can identify trade-offs between metabolites. Furthermore, while tools such as Pareto optimization (Gao et al.), ObjFind, pFBAwEB, and Bayesian-based selection focus on only one aspect, such as addressing objective uncertainties or ranking objectives, SCOOTI integrates all these considerations simultaneously. Nevertheless, methods grounded in rigorous mathematics remain valuable as references or baselines for validating fully or semi-data-driven methods like SCOOTI and GEFMAP. Similarly, BOFdat follows a rigorous protocol for formulating biomass objective functions. However, BOFdat depends heavily on input data quality, does not address multi-objective problems, and cannot formulate non-proliferative objectives, while SCOOTI can (Table 2).

### 2.9. Current Challenges of Inferring Metabolic Objectives Based on Omics Data

The integration of omics data enables the inference of metabolic objectives, but not all data types provide the same level of information for this purpose. Fluxomics data are the most direct and powerful support for objective inference since models can be generated by minimizing the differences between predicted and experimental fluxes. While less direct, time-course metabolomics can also yield good objective inferences. Changes in metabolite levels over time can be converted into estimated fluxes [76], which constrain metabolic models. However, fluxomics and metabolomics data are often less accessible than transcriptomics and proteomics, particularly in studies involving mammalian cells. Additionally, these data types are frequently noisy and limited, with many metabolites or reactions unrecorded.

In contrast, transcriptomics and proteomics are more readily available and can provide sufficient information for constraint-based modeling and statistical analysis. However, using transcriptomics and proteomics for objective inference introduces potential biases due to the assumption of a linear dependency between functional metabolic enzymes and their corresponding RNAs or proteins. This assumption oversimplifies the complexities introduced by post-translational modifications and other regulatory mechanisms. The integration of other data types, such as epigenomics [80], can partially address this issue. The integration of multi-omics data, as demonstrated by tools like SCOOTI, significantly improves objective inference, although the constraint effects from transcriptomics, proteomics, and metabolomics are not equivalent. Assigning appropriate weights to different constraints could enhance inference accuracy.

Single-cell data, while inherently noisy and sparse, offer more detailed information on cell-to-cell variation in metabolism compared to bulk data. High-quality control procedures can substantially improve the quality of objective inferences from single-cell data. Nonetheless, the primary challenge of integrating single-cell data for objective inference relates to computational demands. Considering cellular heterogeneity further exacerbates the computational burden, making high-performance computing essential for single-cell objective inference, as seen with SCOOTI. Grouping and approximation methods, such as COMPASS and SEACells, can help to mitigate these challenges by reducing computational complexity while preserving biological insights [135].

### 2.10. Validation of Inferred Metabolic Objectives

The validation of inferred metabolic objectives is critically important yet challenging. First, there is no consensus on how to formulate metabolic objectives. For instance, both metabolites and reactions can serve as components of metabolic objectives, and non-linear objectives are also proposed as potential candidates. This lack of standardization complicates the validation of inference models due to the absence of consistent references. Second, the lack of curated phenotype-to-metabolic-objective mappings makes precise validation difficult without ground truth data. Third, metabolic objectives may represent combinations of multiple phenotypes. For example, a metabolic objective could comprise 70% growth and 30% maintenance, further complicating validation efforts.

To address these challenges, we propose validation methods spanning four different aspects:

Synthetic data testing: Inference models can first be tested using synthetic data, such as flux predictions with known objective functions. This provides a controlled environment for evaluating model performance.

Component recapitulation: The components of inferred metabolic objectives (e.g., weights of metabolites or reactions) should reflect the metabolic features of a cell. For instance, proliferating cells prioritize biomass accumulation, and so the weight of biomass in the metabolic objective should be the highest.

Gene essentiality analysis: Gene essentiality predictions can be validated experimentally using knockout strains as an independent source of verification for model outputs.

Phenotype similarity: Inferred metabolic objectives should exhibit relative similarity to known phenotypes. For example, growth phenotypes are typically represented by biomass objectives, and the metabolic objectives of a proliferating cell are expected to closely resemble biomass objectives compared to non-proliferative objectives. While some phenotypes lack corresponding metabolic objectives for direct comparison, reference objectives can be accumulated by inferring objectives from cells with well-defined phenotypes, such as migration [136]. Comparing unknown metabolic objectives to this reference dataset could provide insights into relative cellular phenotypes.

Although challenges remain, these strategies provide a foundation for systematically validating metabolic objective inference methods and advancing our understanding of cellular metabolism.

### 2.11. Bioengineering Applications of Cellular Objectives

Various fields of bioengineering, including biomanufacturing, drug development, personalized medicine, and regenerative medicine, can gain from understanding and manipulating cellular objectives. Objectives can help design targeted interventions and optimize biological systems for industrial and therapeutic uses. The identification of objectives can inform strategies to engineer cells to enhance the production of biofuels, pharmaceuticals, or novel biomaterials. Multi-objective optimization frameworks like OptKnock and MOMO use trade-off analysis to improve yields of bioproducts while maintaining cellular viability [106,107]. Applications include the production of biofuels like ethanol, secondary metabolites such as antibiotics, and complex biopharmaceuticals such as monoclonal antibodies. These advancements enable the fine-tuning of resource allocation to maximize target metabolite synthesis while minimizing unintended metabolic burdens.

The field of synthetic biology can benefit from the integration of cellular objectives into the design of novel biosystems. In synthetic circuits, engineered cells can be programmed to prioritize specific phenotypes, such as rapid adaptation to environmental changes or precise metabolite production [137]. Understanding trade-offs between growth and metabolic productivity allows researchers to design robust synthetic systems that function efficiently under industrial conditions [138]. For example, Nilsson and Nielsen demonstrated that constraint-based models’ support for trade-offs between glycolysis and respiration, known as the Crabtree Effect, is caused by ATP synthases in yeast [139]. The authors suggest that the trade-offs between a synthetic pathway and the total protein pool can be identified the same way. These previous studies showed that modeling cellular objectives can be important for helping the design of genetic circuits and strain genomes.

Incorporating cellular objectives into computational models can enhance drug discovery pipelines. Personalized metabolic objectives inferred from patient-specific omics data allow tailored therapeutic strategies, optimizing treatment efficacy while minimizing side effects [140]. For instance, a graph model based on genome-wide association study (GWAS) data linked casual genes and metabolites to clinically relevant phenotypes [141]. Despite the success, metabolic modeling offered more interpretability and outperformed traditional genome-wide association study (GWAS) in predicting phenotypes from genotypes [142].

Personalized metabolic objectives can be used to integrate genetic data. For instance, AGORA2 reconstructed metabolic models for over 7000 microbial species to predict gut microbial responses for a cohort of 616 patients [143]. Foguet et al. also constructed organ-specific metabolic models for more than 520,000 individuals from the INTERVAL and UK Biobank cohorts. These models facilitated a fluxome-wide association study that identified reactions associated with coronary artery disease [144]. While these studies primarily focused on flux solutions, the correlation between fluxes and disease phenotypes hinted at the potential for extracting personalized metabolic objectives. This could enable us to rank the significance of metabolites in relation to a disease phenotype, as well as corresponding coefficients, as inferred by ObjFIND and SCOOTI.

In tissue engineering, cellular objectives can guide the differentiation of stem cells and the maintenance of tissue-specific phenotypes. During embryogenesis or stem cell differentiation, metabolic and transcriptional objectives shift dynamically. Modeling these shifts enables precise control over differentiation pathways, crucial for engineering functional tissues [145]. The integration of cellular objectives into bioengineering applications promises to revolutionize fields spanning biotechnology, medicine, and environmental science. Emerging methods such as SCOOTI- and Pareto-based frameworks offer new opportunities to refine cellular objectives and optimize trade-offs between competing functions. While various metabolic objective inference methods expand the horizons of metabolic modeling approaches, the standardization of user-friendly pipelines and optimization of computational efficiency remains essential in order to facilitate the utilization of customized objectives.

## 3. Conclusions

The exploration of cellular objectives, trade-offs, and archetypes offers a comprehensive framework for understanding how cells regulate and optimize their functions across diverse biological systems. The development of computational methods such as ParTI and SCOOTI has enabled researchers to link high-dimensional omics data to phenotypic outcomes. These tools provide a quantitative foundation with which to model cellular behavior, resource allocation, and adaptive strategies, shedding light on how cells balance competing tasks like growth, maintenance, and differentiation under varying environmental conditions.

Despite these advances, significant challenges persist. The complexity of cellular systems, especially in non-proliferative or specialized cells, makes it difficult to identify universal objectives that fully explain cellular phenotypes. Current models, while effective for proliferative cells such as bacteria and cancer cells, often fall short in accurately predicting the behavior of cells that prioritize maintenance, differentiation, or other specialized functions over biomass production. Furthermore, the non-linear and dynamic interactions between transcriptional, proteomic, and metabolic networks complicate the task of linking gene expression patterns directly to metabolic activity and phenotype.

Future research should focus on improving computational methods for multi-objective optimization, refining the accuracy of inferred objectives and developing user-friendly pipelines that allow the broader application of these models in personalized medicine, drug development, and bioengineering. By exploring the dynamic interplay between transcriptional programs and metabolic activity, we can gain a deeper understanding of how cells navigate complex biological landscapes to maintain functionality and fitness across diverse environments.

## Figures and Tables

**Table 2:** Methods to infer metabolic objectives/tasks via GEMs.

Name	Input	Output	Summary	Organisms	Ref.
ObjFIND	Fluxomics	Objective functions	Bi-layer optimization problems firstly minimizing the distance between predicted and experimental fluxes to solve coefficients of candidate demand reactions	Bacterial cells	[90]
BOSS	Fluxomics	Objective functions	Bi-layer optimization problems firstly minimizing the distance between predicted and experimental fluxes to solve coefficients of randomly scanned objective functions	Bacterial cells	[91]
SEED	Fluxomics	Objective functions	Approximate coefficients with experimental data and select biomass components with a template in which non-universal metabolites were chosen when meeting certain criteria	Bacterial cells	[86]
invFBA	Fluxomics	Objective functions	Two-step optimization problems that minimize the error between measured and predicted objective functions	Bacterial cells	[92]
Bayesian-based selection	Fluxomics	Score of objective functions	Score each candidate objective functions with probabilities calculated based on a Bayesian-based function of measured fluxes	Bacterial cells	[93]
BOFdat	Multiomics	Objective functions	Draft coefficients of biomass components based on omic datasets and phenotypes (e.g., growth) measurements and finalize the functions with genetic algorithms	Bacterial cells	[110]
BTW and HIP	Fluxomics	Objective functions	BTW weighs multiple pre-built objective functions to fit phenotype measurement such as growth rate and HIP interpolates between different biomass compositions	Bacterial cells	[88]
pFBAwEB	Transcriptomics, proteomics, and fluxomics	Ensemble representations of biomass	Gather coefficient of variation to generate a range of biomass composition	*E. coli*, *S. cerevisiae*, and CHO cells	[89]
Gao et al.	Transcriptomics	Metabolic tasks	Leverage ParTI method to identify metabolic tasks from transcriptomics datasets and predicted fluxes and phenotypes corresponding to the metabolic tasks	Cancer cells	[129]
CellFile	Transcriptomics	Scores of metabolic tasks	Summarize flux solutions of transcriptomics-based context-specific models with metabolic subsystems	Mammanlian cells	[128]
GEFMAP	Single-cell transcriptomics	Objective function and scores	GEFMAP-constructed graph neural network from single-cell data and the association between gene to infer objective functions	*E. coli*, *S. cerevisiae*, and hESC	[127]
SCOOTI	Single-cell and bulk multiomics	Metabolic objectives	Infer condition- or cell-specific metabolic objectives based on any type of omics dataset and identifies metabolic traits with these objectives	Mammanlian cells	[126]

## Data Availability

No new data were created or analyzed in this study.
